# Three Cases of Inoculation with the Variolæ Vaccinæ

**Published:** 1799-08

**Authors:** R. Redfearn

**Affiliations:** Lynn, Norfolk


FOR THE MEDICAL AND PHYSICAL JOURNAL.

### *Three Cases of Inoculation with the Variolæ Vaccinæ.*

[Communicated by R. Redfearn, M.D. Lynn, Norfolk.]

### Case I.

On the 20th of March, 1799, a boy named Ripper, aged three years and a half, being rather of a groſs habit of body, was inoculated under my direction by Mr. Crawforth, jun. ſurgeon, with vaccine matter, which I had received the day before from Dr. Pearson, of Leiceſter-ſquare, London. A ſmall inciſion was made upon the left arm, in the uſual place of inoculation for the variolous infection; into this was inſerted a piece of thread, thoroughly impregnated with the aforeſaid matter, moiſtened with ſteam, and ſecured in a proper manner with ſticking-plaſter, and alſo a bandage to prevent its removal.

Monday, March 25th, being the ſixth day after inoculation, upon removing the bandage and ſticking-plaſter from the inciſion, it appeared to be elevated, and in a ſtate of inflammation, attended with ſmall irritated eminences in its vicinity; but no conſtitutional illneſs from this local action had hitherto ſupervened. However, the following day, being the ſeventh after inoculation, upon re-examining the inoculated part, the inflammation and ſwelling were found to be much increaſed. A ſmall flat veſication was alſo obſerved in the centre of the inflamed part, the boy being now ſeized with fever, viz. rigors, fluſhings of the face, quick pulſe, head-ach, ſkin hot and dry, and accompanied with conſiderable languor and drowſineſs. Thoſe febrile ſymptoms continued during the four following days (the 27th, 28th, 29th, and 30th of March), being eleven days after inoculation, and five from the commencement of the fever. At this period, an eruption appeared upon the face, hands, and back, although not more than forty puſtules were found upon the whole ſurface of the body.

Monday, April 1ſt (being the thirteenth day), the eruption had increaſed in ſize, but not in quantity. On the appearance of the puſtules, the fever ſubſided, and two or three very fine circular, elevated, flat puſtules were alſo obſerved near the inoculated part, exhibiting to the eye ſo beautiful a poliſh, that a piece of thread was thoroughly impregnated with matter taken from them, and reſerved for future inoculation.

Friday, April 5th (the ſeventeenth day), the eruption in many parts of the body ſeemed to be dying away, and a poliſhed dark brown ſpot was viſible in the middle of the puſtules. The inoculated part was much contracted in ſize,

ſize, and a flat brown incruſtation was obſerved in its centre. In all other reſpects the boy appeared to be perfectly well.

Case II.—A girl, aged eleven months, labouring under dentition, *unweaned*, and rather of a delicate conformation, was inoculated in a ſimilar way as the preceding patient, on the 20th of March, and with a piece of the ſame thread, impregnated with vaccine matter, as before deſcribed.

Sixth day, Monday, March the 25th, the inoculated part was a little inflamed and tumid: no pain of the axilla, and free from every ſymptom of conſtitutional indiſpoſition.

The eighth day, Wedneſday, March 27th, the inflammation upon the inoculated part was rather more increaſed, comparatively with its appearance, than on the ſixth, and ſome ſlight febrile action was alſo obviouſly manifeſt.

On the eleventh day, March 30th, an eruption appeared upon the face, neck, hands and legs, extending itſelf alſo over the whole ſurface of the body, and the patient was extremely reſtleſs and uneaſy.

Sixteenth day, April 4th, the fever vaniſhed ſoon after the commencement of the eruption. The latter now aſſumed a puſtular form, and was perfectly analogous to the variolous diſeaſe. The palpebræ were quite cloſed, the eruption being in every reſpect as copious as generally happens in the uninoculated ſmall-pox.

Seventeenth day, April 5th, the puſtules were advancing rapidly to a ſtate of ſuppuration, and appeared upon many parts of the body to be very circular, prominent, and full of matter. The palpebræ were not ſo much cloſed as on the preceding day.

Twentieth day, Monday, April 8th, on many parts of the body the puſtules were dying away, particularly upon the face; and a dark, browniſh ſpot, of a horny contexture, and gloſſy hue, was obſervable upon their apex. They were, indeed, ſo remarkably diſtinct and beautiful on the lower extremities, that I was induced to have a drawing taken of them by Mr. Butcher, of Yarmouth, an eminent portrait and landſcape-painter.

Case III.—Monday, April 4th, a girl of the name of Partridge, aged three years and a half, and of a full habit of body, was inoculated with matter taken from the boy, Ripper, on the thirteenth day of the diſeaſe, as has been already mentioned in the firſt caſe.

On the ſixth day after inoculation (April 6th), there appeared a redneſs round the edges of the inciſion upon the inoculated arm, but no elevation or

ſwelling

ſwelling was obſervable. The patient remained free from every ſymptom of fever.

Seventh day, April 7th, the edges of the inoculated part were rather more inflamed than on the preceding day. The patient complained of head-ach, chillineſs, and fluſhings, with other ſymptoms of fever, manifeſtly ariſing from abſorption of vaccine matter into the ſyſtem, from the inoculated part. No pain of the axilla.

Tenth day, April 10th, the fever had continued ſince the ſeventh day, but in ſo ſlight a degree, that the child ran about the houſe as uſual, making little or no complaint. However, at this period of the diſeaſe, being ten days after inoculation, and four from the commencement of the fever, a few eruptive ſpots were obſerved upon the face and hands, not more in number than five or ſix. When this took place, the fever almoſt immediately ſubſided; theſe ſpots made little progreſs in ſize, and died away in the courſe of a few days, having a darkiſh ſcab in their centre, as they evidently had contained nothing but lymph.

The three preceding caſes of the cow-pox have been inoculated a ſecond time with variolous matter, taken from the human ſyſtem, and a greater quantity was introduced than is uſually done on ſuch occaſions: but no morbid action, either local or general, ever commenced. Their arms were examined with the greateſt attention on the ſeventh, eighth, tenth, and twelfth days after the variolous matter was inſerted, yet the leaſt diſcolouration of the inciſions could not be perceived; and on the laſt-mentioned day they appeared to be quite well, and the inciſions were perfectly healed.

